# Imaging of ^212^Pb in mice with a clinical SPECT/CT

**DOI:** 10.1186/s40658-023-00571-6

**Published:** 2023-08-21

**Authors:** Monika Kvassheim, Anna Julie Kjøl Tornes, Asta Juzeniene, Caroline Stokke, Mona-Elisabeth R. Revheim

**Affiliations:** 1 Division of Radiology and Nuclear Medicine, Department of Physics and Computational Radiology, Oslo University Hospital, Oslo, Norway; 2 Faculty of Medicine, University of Oslo, Oslo, Norway; 3 Department of Radiation Biology, Institute for Cancer Research, Oslo University Hospital, Oslo, Norway; 4 ARTBIO AS, Oslo, Norway; 5 Department of Physics, University of Oslo, Oslo, Norway; 6 Division of Radiology and Nuclear Medicine, Department of Nuclear Medicine, Oslo University Hospital, Oslo, Norway; 7 The Intervention Centre, Division of Technology and Innovation, Oslo University Hospital, Oslo, Norway

**Keywords:** Lead-212, ^212^Pb, Alpha, Therapy, Imaging, SPECT, Preclinical, Phantom

## Abstract

**Introduction:**

^212^Pb is a promising radionuclide for targeted alpha therapy. Here, the feasibility of visualising the tumour uptake and biodistribution of ^212^Pb-NG001 in mice with a clinical SPECT/CT scanner was investigated.

**Methods:**

A mouse phantom with ^212^Pb was imaged with a clinical- and a preclinical SPECT/CT scanner. Different acquisition and reconstruction settings were investigated on the clinical system (Siemens Symbia Intevo Bold). Two athymic nude mice carrying PC-3 PIP prostate cancer tumours of 235–830 μl received 1.44 MBq of ^212^Pb-NG001 and were imaged 2, 6, and 24 h post-injection on the clinical SPECT/CT with a Medium Energy collimator and a 40% energy window centred on 79 keV. All acquisition times were 30 min, except the mouse imaging 24 h post-injection which was 60 min. After the final imaging, the organs were harvested and measured on a gamma counter to give an indication of how much activity was present in organs of interest at the last imaging time point.

**Results:**

Four volumes in the mouse phantom of ~ 300 μl with 246–303 kBq/ml of ^212^Pb were distinguishable on images acquired with the clinical SPECT/CT with a high number of reconstruction updates. With the preclinical SPECT, the same volumes were easily distinguished with 49 kBq/ml of ^212^Pb. Clinical SPECT/CT images of the mice revealed uptake in tumours and bladders 2 h after injection and in tumours containing down to approximately 15 kBq/ml at 6 and 24 h after injection.

**Conclusion:**

Although the preclinical scanner should be used preferentially in biodistribution studies in mice, the clinical SPECT/CT confirmed uptake in small volumes (e.g. ~ 300 μl volume with ~ 250 kBq/ml). Regardless of system, the resolution and sensitivity limits should be carefully determined, otherwise false negative or too low uptakes can be wrongly interpreted.

## Introduction

^212^Pb decays to ^212^Bi via beta emission with a half-life of 10.64 h. As ^212^Bi only emits one alpha particle per decay to stable ^208^Pb, either directly in its decay to ^208^Tl (36%) or through its relatively short lived daughter ^212^Po (64%), daughter redistribution is not as much of a concern as for other alpha emitters proposed for therapy [1]. Factors such as a convenient half-life and industrial scale production methods also contribute to making ^212^Pb a promising radionuclide for targeted alpha therapy [2]. In addition, it offers potential for a theragnostic approach, as ^203^Pb is easily imaged and can be used as a surrogate radionuclide for patient selection, treatment monitoring, and dosimetric estimation [3–5]. Direct imaging of ^212^Pb is also possible, since gamma photons of 238.6 keV and X-rays of 75–91 keV, with intensities of 43% and 36%, respectively, are emitted during the decay to ^212^Bi [1]. Imaging of alpha emitters is often considered challenging, due to low yields of photons with energies suitable for imaging, low administered activities, and high energy gamma emissions causing scatter [6]. For ^212^Pb, there is a reasonable photon yield, and since the half-life is shorter than for instance ^225^Ac, ^223^Ra, and ^227^Th and there is only one alpha particle emitted per decay chain; in contrast to other alpha emitters considered for therapy, more activity can be administered. There are still high energy emissions causing scatter and noise, in particular a 2.6 MeV emission from ^208^Tl [7], but imaging of ^212^Pb might be more easily achievable than for other alpha emitters. Planar scintigraphy images of ^212^Pb in patients [8] and in monkeys [9] have been acquired and a recent phantom study showed that quantitative SPECT/CT imaging of ^212^Pb could be feasible [10].

Whilst biodistribution studies in mice are well established, SPECT imaging offers a non-invasive alternative that allows for visualisation of the biokinetics in a mouse over time [11]. Preclinical SPECT scanners are used by many developers of radiopharmaceuticals, but some institutions might only have clinical scanners available, which provided the motivation to determine if clinical SPECT/CTs could be used for imaging mice injected with ^212^Pb. In this study, the prostate specific membrane antigen (PSMA) targeting radioligand ^212^Pb-NG001 [12] was imaged in mice to investigate the potential for small volume imaging with the clinical SPECT/CT. The aim of the study was to evaluate whether imaging the distribution of ^212^Pb in mice was achievable and to determine the most promising imaging protocol. To achieve this, a mouse phantom was imaged with both a preclinical and a clinical SPECT/CT. After, two mice injected with 1.44 MBq ^212^Pb-NG001 were imaged on the clinical system.

## Method

### Preparation of ^212^Pb and radiolabelling with NG001

The ^212^Pb was obtained from gaseous ^220^Rn emanated from a decaying ^228^Th source using a generator described by Li et al. [13]. Radiolabelling of the small molecule PSMA targeting ligand, NG001, quality control, and radioactivity measurements was performed as described previously [14].

### Imaging protocols on the clinical scanner

Four imaging protocols were examined with a mouse phantom to guide the choice of protocol for mouse imaging. A Siemens Symbia Intevo Bold SPECT/CT with a 3/8″ crystal was used. Two collimators, High Energy (HE) and Medium Energy (ME), and two energy windows, 40% at 79 keV and 20% at 239 keV, were combined in the four protocols. Dual scatter windows of 20% for the 79 keV peak and 5% for the 239 keV peak were used for scatter correction. Images using different protocols were acquired separately, and each acquisition was 30 min. The SPECT images were acquired with body-contouring orbits, a 256 × 256 matrix, and 60 views with acquisition during steps. SPECT images were reconstructed with Flash-3D, with both 30 iterations and 4 subsets (30 × 4) and 30 iterations and 30 subsets (30 × 30). Attenuation correction based on the CT image was performed using the central energy of the energy window. The voxel size was 14 mm^3^ (2.4 × 2.4 × 2.4 mm^3^). No filtering was applied.

The ME collimator and a 40% energy window at 79 keV with 20% dual scatter windows were chosen for SPECT/CT imaging of the mice. The mice were imaged for 30 min 2 and 6 h post-injection and for 60 min 24 h post-injection. The images were reconstructed with the 30 × 4 reconstruction.

### Imaging protocols on the preclinical scanner

The VECTor 6 scanner (MILabs, The Netherlands) was used to image the mouse phantom. The 20% energy window centred on 239 keV studied on the clinical scanner was investigated again with the VECTor system, with the same dual scatter windows. A high sensitivity pinhole collimator (XXUHS) was used. The images were acquired for 30 min. Images were reconstructed with the POSEM algorithm, with 30 iterations and 2 subsets, with attenuation correction applied. Images were reconstructed without filters and with a 2.4 mm filter. The voxel size was 0.008 mm^3^ (0.2 × 0.2 × 0.2 mm^3^).

### Mouse phantom

A mouse phantom from BIOEMTECH (BIOEMTECH, Greece) with fillable organs and tumours was used to prepare for scanning of mice on the clinical SPECT/CT scanner and to compare collimators and energy windows. For imaging on the clinical scanner, to simulate the expected biodistribution of the mice, a high-activity concentration of 363 kBq/ml at preparation was put in the tumour (0.30 ml, 109 kBq), kidneys (0.69 ml, 250 kBq), and bladder (0.29 ml, 105 kBq). A low-activity concentration of 46 kBq/ml was used in the brain (0.17 ml, 7.8 kBq), heart (0.28 ml, 13 kBq), and liver (1.16 ml, 53 kBq). The high-activity concentration was 246–303 kBq/ml during the acquisitions, and the low-activity concentration was 30–37 kBq/ml.

The phantom was also imaged on the preclinical SPECT, to put the results with the clinical SPECT into the context of what is achievable with dedicated scanners. Prior to imaging with the preclinical scanner, the mouse phantom was filled again with a high-activity concentration solution of 350 kBq/ml in the tumour (0.30 ml, 105 kBq), kidneys (0.70 ml, 245 kBq), and bladder (0.29 ml, 102 kBq). A low-activity concentration of 40 kBq/ml was placed in the brain (0.17 ml, 6.8 kBq), heart (0.28 ml, 11.2 kBq), and liver (1.16 ml, 46.4 kBq). The phantom was imaged once with the high-activity concentration 241 kBq/ml and the low-activity concentration 27.5 kBq/ml, and once 24.6 h later with the high-activity concentration 48.5 kBq/ml and the low-activity concentration 5.5 kBq/ml.

### Animal and tumour xenografts

Two male Hsd: Athymic Nude-Foxn1^nu^ mice bred at the Department of Comparative Medicine at the Norwegian Radium Hospital (Oslo University Hospital, Oslo, Norway) were used in the study. The animal experiments were approved by the Institutional Committee on Research Animal Care and the Norwegian Food Safety Authority (Brumunddal, Norway, approval: FOTS ID 22197).

The mice were inoculated subcutaneously in both flanks with 5 × 10^6^ PC-3 PIP prostate cancer cells/flank in RPMI1640 medium without supplements mixed 1:1 with Matrigel Matrix (Corning Inc., Corning, NY, USA). When tumours were 235–830 mm^3^, the mice were administered intravenously with 1.44 MBq of ^212^Pb-NG001 in 200 µl and imaged as described above. The mice weighed 29.1 g (Mouse A) and 32.5 g (Mouse B) at the day of injection. The mice were anesthetised before each image acquisition by subcutaneous injection of 7–8 mg/kg of xylazine (Rompun, Bayer, Istanbul, Turkey), 0.2 mg/kg butorphanol (Torbugesic®, Zoetis, Canada Inc., Canada), and 4.4–5.0 mg/kg of zolazepam and tiletamine (Zoletil, Virbac, Glattbrugg, Switzerland). Simplex eye ointment (Ophtha AS, Gentofte, Denmark) was used to prevent dryness of the eyes. Anesthetised mice were positioned on the SPECT patient bed surrounded by heating sources to maintain stable body temperature during imaging, following euthanasia after the final imaging acquisition.

Organs (blood, urine, testes, prostate, salivary gland, tumours, skin, kidneys, liver, spleen, small intestine, large intestine, stomach, lungs, heart, bladder, femur, muscle, brain, and skull) were harvested and measured in a gamma counter as described by Stenberg et al. [14].

## Results

**Fig. 1:**
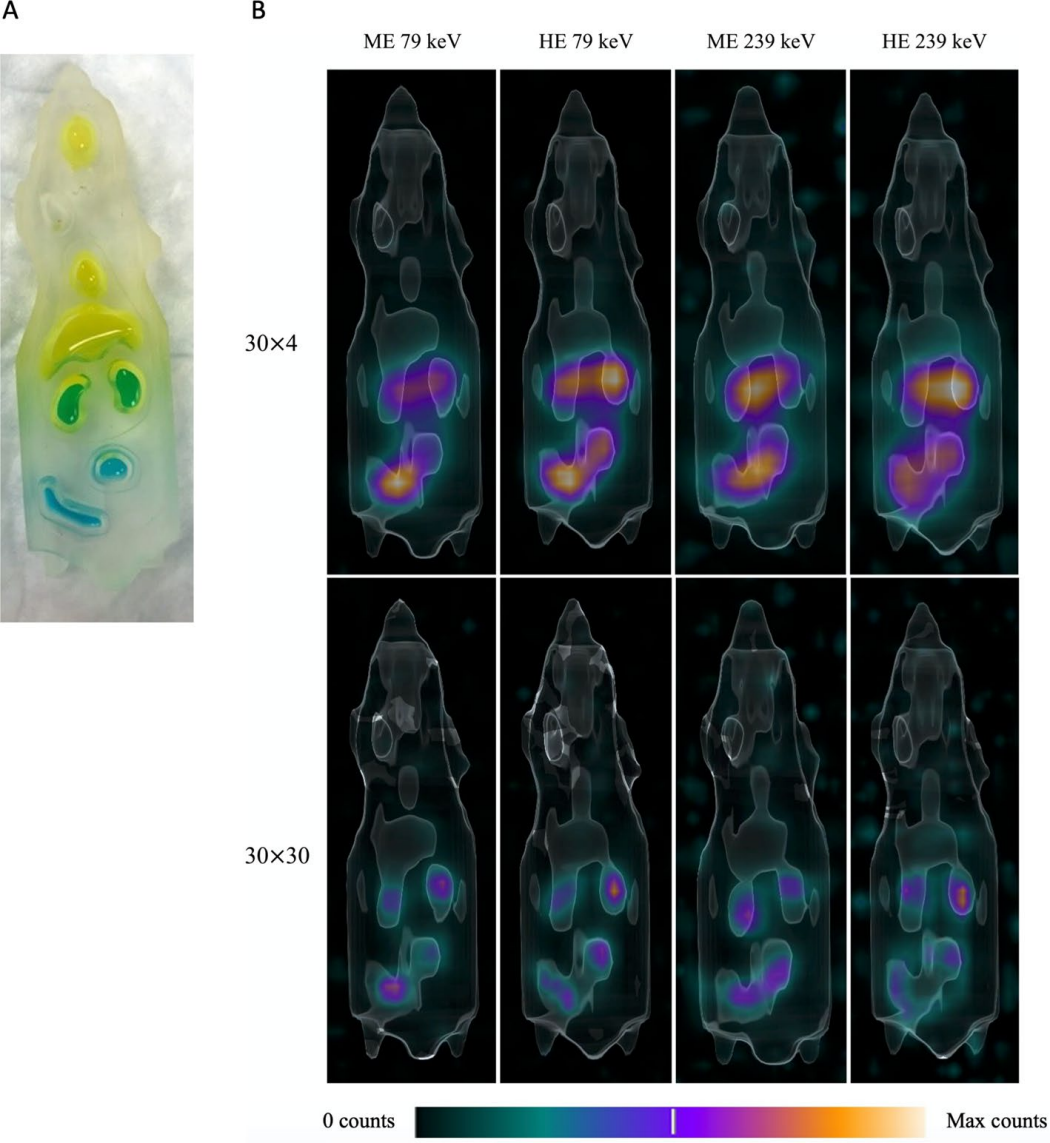
A mouse phantom without the lid after it was filled with ^212^Pb is shown in panel (**A**). The high-activity concentration solution (363 kBq/ml) was coloured blue (kidneys appear green) and the low-activity concentration solution (46 kBq/ml) was coloured yellow. Panel (**B**) shows maximum intensity projection SPECT/CT images of the mouse phantom in the anterior view acquired with the clinical SPECT/CT. The top row shows the 30 × 4 reconstruction, and the bottom row shows the 30 × 30 reconstruction. The activity concentrations in the high activity regions were 303 kBq/ml, 291 kBq/ml, 256 kBq/ml, and 246 kBq/ml for ME 79 keV, ME 239 keV, HE 79 keV, and HE 239 keV, respectively

A picture of the phantom is shown in Fig. 1A, with the high-activity concentration regions in blue and the low-activity concentration regions in yellow. Figure 1B displays maximum intensity projection images from the clinical SPECT/CT of the mouse phantom filled with ^212^Pb for the four imaging protocols. The four high-activity concentration regions could be distinguished with a high number of iteration updates. The image quality was similar between the four studied acquisition protocols, but as the sensitivity was higher with the 79 keV energy window, this was chosen for the mouse imaging. There was no significant difference between the collimators, and hence, the ME collimator was chosen due to the shorter hole length.

**Fig. 2:**
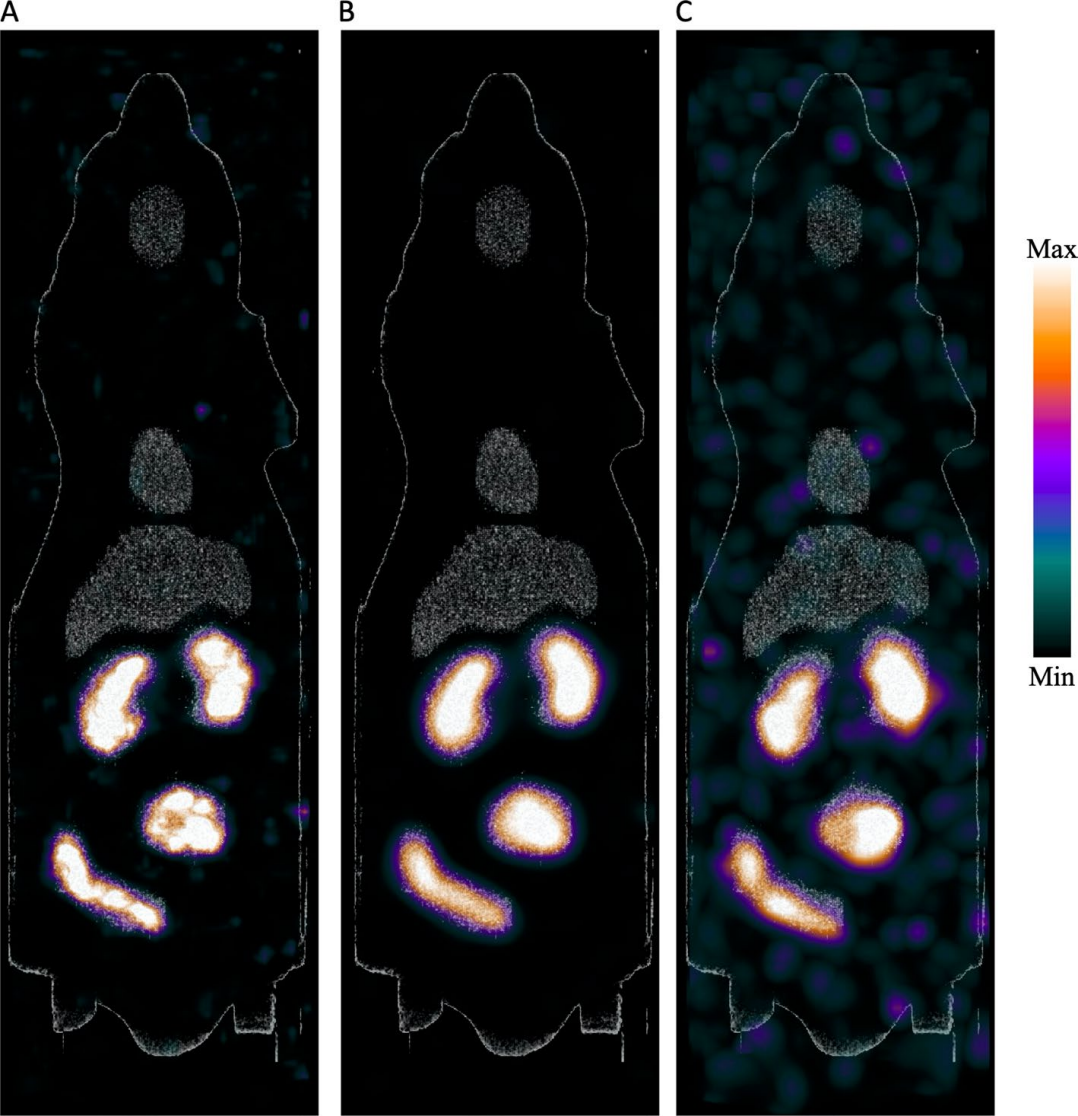
Maximum intensity projection images of the mouse phantom acquired with the preclinical SPECT/CT. Image (**A**) shows the highest activity concentration imaged, 241 kBq/ml, similar to the activity concentration when the phantom was imaged on the clinical SPECT, without filters applied. Image (**B**) is from the same acquisition, but with a 2.4 mm filter applied. Image (**C**) is from an acquisition 24.6 h later, with 49 kBq/ml in the high-activity concentration volumes, with a 2.4 mm filter applied

The four volumes in the mouse phantom with high-activity concentrations were clearly visible and distinguishable on images from both acquisitions with the preclinical SPECT/CT, but artifacts were more prominent for the later time point (Fig. 2). Even at the later time point with 48.5 kBq/ml in the high-activity concentration volumes, the volumes were more easily discernible than those obtained with the clinical SPECT/CT. When optimising the window settings by adjusting the max of the colour scale to avoid the high-activity concentration regions hiding lower activity concentrations, the low-activity concentration volumes with 27.5 kBq/ml could also be visualised.

**Fig. 3:**
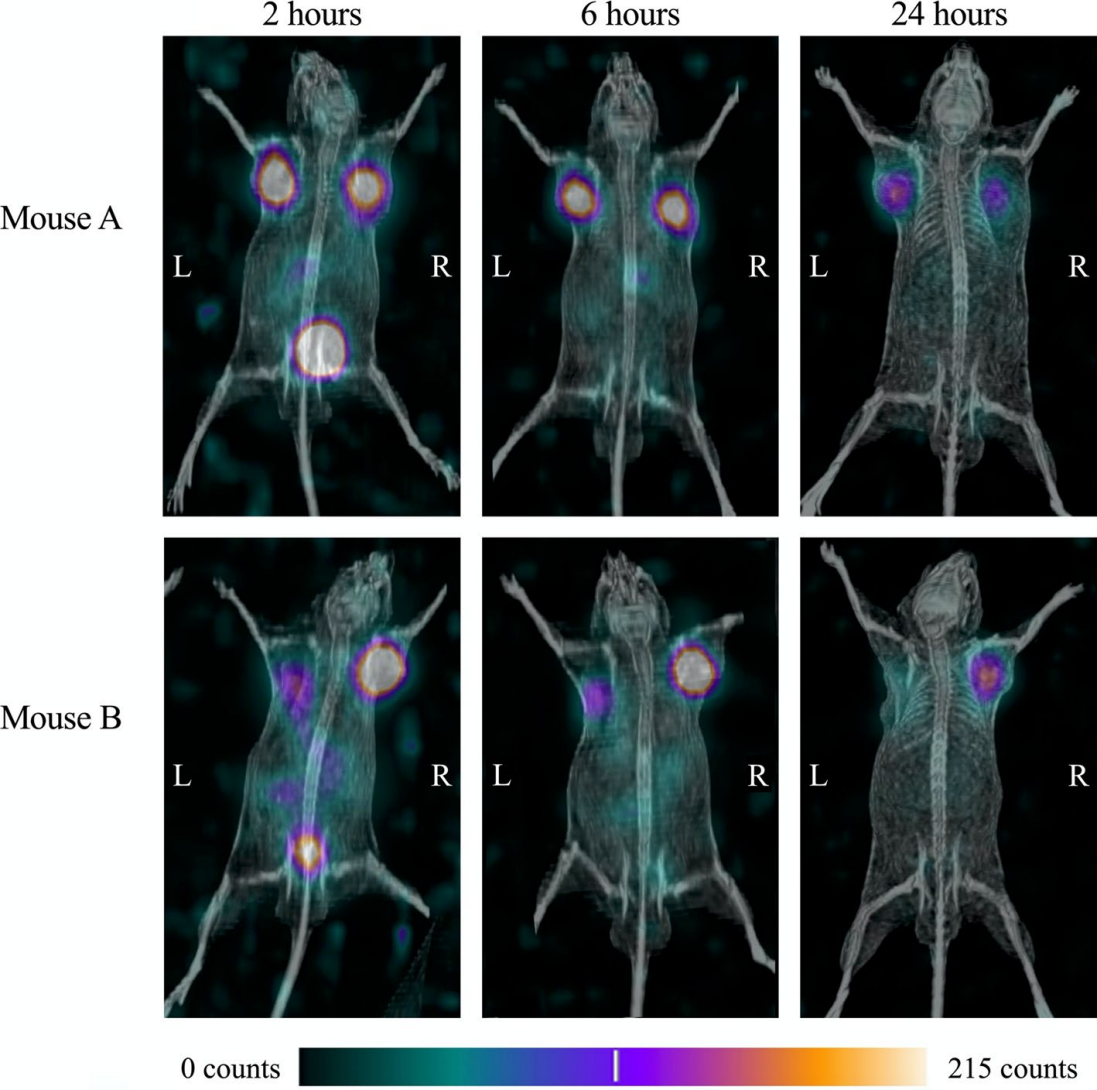
Maximum intensity projection SPECT/CT images of Mouse A (upper) and Mouse B (lower) in the anterior view acquired with the clinical SPECT/CT. The mice were imaged 2, 6, and 24 h post-injection with 1.44 MBq of ^212^Pb-NG001; the acquisition time was 60 min for the last time point and 30 min for the first two

In the images of mice acquired with the clinical SPECT/CT scanner, uptake of ^212^Pb-NG001 in the four tumours was visible at all three time points, and uptake in the bladders was observed after two hours (Fig. 3). A slight uptake was also observed in the kidney and liver region at all time points when adjusting the image settings, but the signal was weak compared to that of tumours and bladders. The biodistribution study showed that the majority of uptake of ^212^Pb-NG001 at 24 and 26 h post-injection was in the tumours, consistent with the SPECT/CT images, but also that there was uptake in the kidneys and livers (Table 1).

**Table 1:** Measured mass and activity of ^212^Pb in tumours, kidneys, livers, and other organs^+^

Organ		Sample mass (g)	Activity* (kBq)	Activity*/mass (kBq/g)
Right tumour	Mouse A Mouse B	1.1 0.7	17.2 20.2	15.6 28.9
Left tumour	Mouse A Mouse B	0.9 0.5	16.2 11.7	18.0 23.4
Kidneys (both)	Mouse A Mouse B	0.4 0.5	2.7 4.0	6.8 8.0
Liver	Mouse A Mouse B	1.4 1.8	3.2 3.1	2.3 1.7
Other^+^	Mouse A Mouse B	2.9 1.8	0.6 0.3	0.2 0.2

*Given at 25.8 h post-injection for Mouse A and 24.4 h post-injection for Mouse B

^+^Other includes blood, urine, testes, prostate, salivary gland, skin, spleen, small intestine, large intestine, stomach, lungs, heart, bladder, femur, muscle, brain, and skull

## Discussion

The feasibility of imaging the activity distribution of ^212^Pb in small mouse volumes with a clinical SPECT/CT was investigated in this study. Images of a mouse phantom were acquired with a clinical- and a preclinical scanner. Four activity regions of approximately 0.3 ml could be distinguished on the images from the clinical scanner with 246–303 kBq/ml of ^212^Pb. As expected, images acquired on the preclinical scanner had much better visual performance for the same volumes, even with a lower activity concentration. However, the volumes containing 27.5 kBq/ml or 30–38 kBq/ml were difficult to discern on both the clinical and the preclinical images. Activity uptake in the tumours and the bladders of the mice was clearly visualised, but the resolution of the clinical system limits the potential to determine smaller volumes and volumes with low-activity concentrations.

Uptake in the kidneys and livers in the mice was not clearly defined above the noise on the images from the clinical scanner. This was not due to negligible uptake in the kidneys and livers, as can be seen in Table 1, but was more likely due to the limited amount of activity in these tissues as well as their small size. There was 3 kBq in the livers after the final imaging time point, and the samples were larger than any of the tumour samples, but they contained around tenfold less activity per gram than the tumours (Table 1). Hence, if the goal is to determine whether there is uptake of a ^212^Pb-based radiopharmaceutical in a mouse kidney or liver, the clinical scanner might be a poor choice, depending on the amount of activity and the biodistribution. Either a preclinical scanner must be used (but smaller mouse organs are likely under the detection limit for the preclinical system with low-activity concentrations of ^212^Pb) or dissection biodistribution studies must be performed. However, if the aim of the study is to establish uptake in the tumours, the clinical scanner could be satisfactory.

**Fig. 4:**
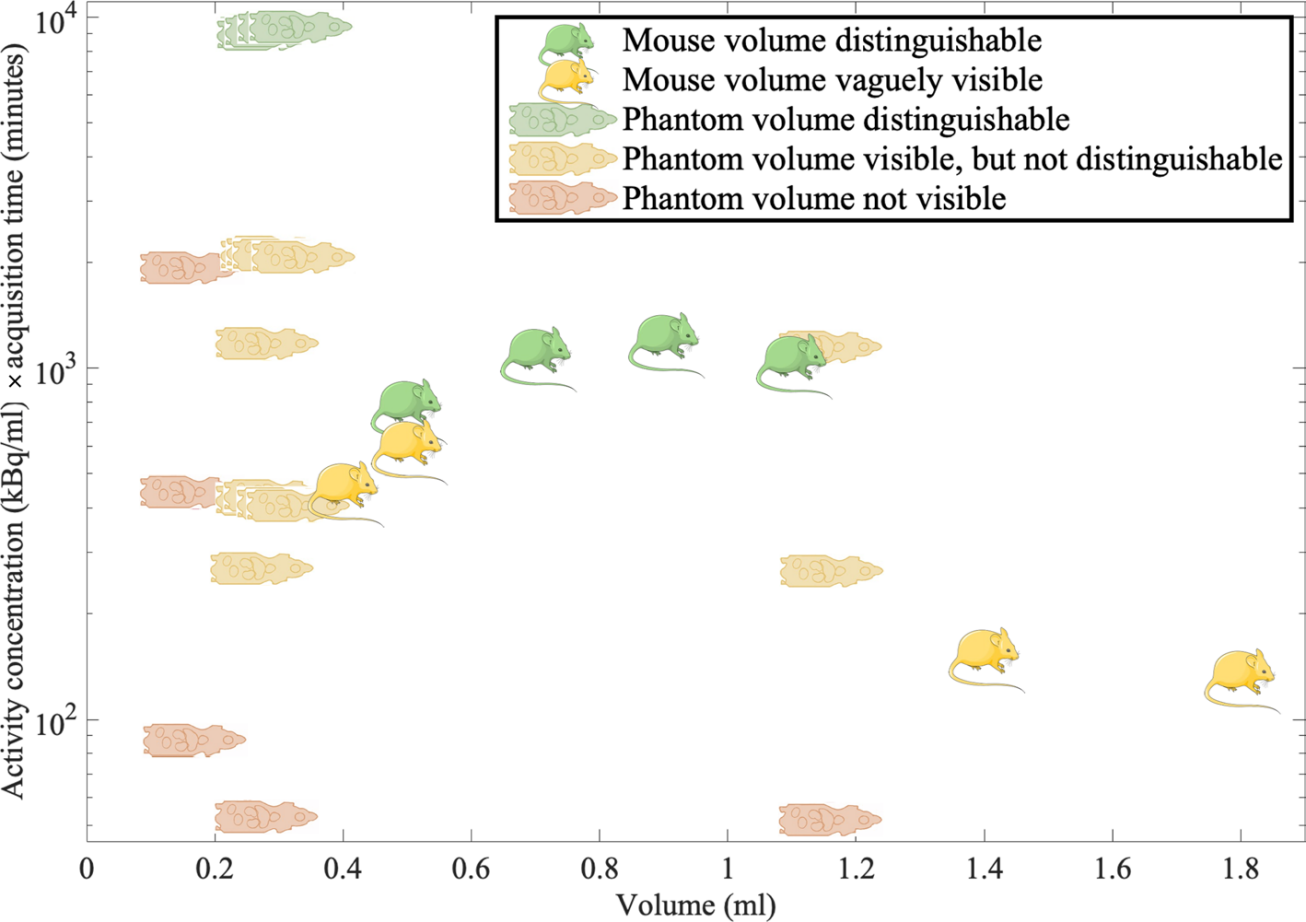
The illustration indicates volumes for which accumulation of ^212^Pb could be visualised and distinguished. It shows how visualisation depends on several factors, here factors volume, acquisition time, and activity concentration are plotted. Many more factors affect which volumes are seen in images, for instance acquisition and reconstruction settings and surrounding volumes with uptake. What is visible in an image is of course objective, but the figure serves to indicate what was found in this study. The mouse symbols indicate volumes seen in the mice, for instance the two yellow mice with the largest volumes represent the liver uptakes of the mice 24 h post-injection. Uptake could be seen with optimised windowing, but they were not clearly distinguishable volumes. The four green mice represent the uptake in tumours after 24 h, all of which were clearly distinguishable. The other symbol represents volumes in the mouse phantom. Only the high-activity concentration regions at the first imaging time point were clearly visible and distinguishable, but some low-activity regions, the heart and liver, could be vaguely made out with optimised settings. The schematic mouse in the figure was provided by Servier Medical Art (https://smart.servier.com/) and the schematic phantom was adapted with permission from a schematic from Bioemtech

Figure 4 illustrates the factors affecting visualisation of volumes of ^212^Pb with the clinical SPECT/CT with the imaging protocol used to image the mice. Some of the factors influencing what can be visualised in SPECT images are shown in the figure; volume, acquisition time, and activity concentration, but other factors also affect which volumes are seen in images; for instance, acquisition and reconstruction settings and surrounding volumes with uptake. What is visible in an image is of course objective, but the figure serves to indicate what was found in this study. The mouse symbols indicate volumes seen in the mice on the images from 24 h post-injection. The other images of the mice are not included in the figure, since we do not have estimates of the activity concentrations at those time points. The two yellow mice with the largest volumes represent the liver uptakes of the mice 24 h post-injection. The two yellow mice with the smallest volumes represent the kidney uptakes of the mice 24 h post-injection. Uptake could be seen with optimised windowing, but they were not clearly distinguishable from each other. The four green mice represent the uptake in tumours after 24 h, all of which were clearly distinguishable. The other symbol represents volumes in the mouse phantom, for which the activity concentration was known at all time points. Only the high-activity concentration regions at the first imaging time point were clearly visible and distinguishable and are shown in green, but some low-activity regions, the heart and liver, could be vaguely made out with optimised settings and are shown in yellow. Other volumes in the mouse phantom, such as the brain or the heart at later time points, could not be visualised at all and are shown in red.

Although imaging mice and a mouse phantom is very different from imaging patients, this study gives an indication of the size of volumes and activity concentrations we might be able to distinguish in patients injected with ^212^Pb-conjugates. However, attenuation and scatter will play a much larger part for patients, especially using an energy window centred on 79 keV. Attenuation of the X-rays will cause the count rate to decrease for patients compared to mice, and as that occurs, more noise can be expected in the images. This will impair the ability to distinguish true activity regions. An additional effect to be aware of is that in patients, larger volumes of uptake are likely to disguise uptake in nearby small volumes due to intensity diffusion or partial volume effects. Our group has previously performed phantom studies with ^212^Pb and discussed image quality and detectability for patients [10].

In relation to a preclinical setting, the preclinical scanner gave superior results compared to the clinical scanner. If a preclinical SPECT is available, it should be used in preference to a clinical SPECT when the purpose is to investigate the biodistribution in mice. Imaging mice in a clinical scanner is challenging for several reasons. For example, in a machine designed for humans, there is no set up for keeping the mice warm during the acquisition; the collimators and detectors have spatial resolutions that are not sufficient to resolve the smallest volumes of interest in a mouse, and the wide bed hinders the detectors getting close to the mice from the side. There are however many advantages to imaging a mouse over imaging a patient which preclinical scanners exploit, for example the low amount of attenuating and scattering material, small volumes that can be enlarged using pinhole collimators, and the ability to bring the detectors close to the volumes of interest.

For SPECT imaging to replace dissection biodistribution studies in mice, the uptake in organs at risk must be quantified. With the clinical SPECT, the kidneys of the mice were here considered too small to quantify the activity in them, but uptake in tumours might be quantifiable. Quantitative imaging with ^212^Pb is possible, but a previous study showed that when there was less than 1 MBq in the field of view of the detectors, the calibration factors became unstable for the system used in this study [10]. However, the instability in the calibration factors observed at low activities was not irregular, but there was a consistent increase in the calibration factors as the activity decreased. To obtain quantitative results with mice on the clinical scanner, calibration factors would need to be acquired with similar geometries and activity levels, using the same acquisition protocol. This could be achieved with the mouse phantom. Regardless, quantification of uptake in small mouse volumes with the clinical SPECT will probably involve large uncertainties from segmentation and pixilation, in addition to the need for a set of well characterised calibration factors and recovery coefficient curves for a range of relevant volumes. Although not investigated here, quantitative imaging might be possible for low-activity concentrations of ^212^Pb on the preclinical scanner, and associated uncertainties are probably smaller than for the clinical scanner. However, partial volume effects, i.e. the loss of reproduced image signal for smaller volumes, are likely to be found, and recovery coefficients to account for such should be established.

In conclusion, images of ^212^Pb from the clinical scanner enabled identification of volumes of 0.3 ml with an activity concentration of approximately 250 kBq/ml, and tumours of approximately 0.7–1.1 ml with an activity concentration of approximately 15–30 kBq/ml (with twice the acquisition time). Thresholds for resolving structures for activity concentration or volume were not determined in this study. As indicated by the illustration in Fig. 4, visibility of volumes will depend on both size and amount of activity contained, as well as technical settings (e.g. acquisition parameters) and physiological factors (e.g. uptake in surrounding tissues). Whilst an indication of the distribution of ^212^Pb in mice was obtained with the clinical SPECT/CT, visualisation of potential uptake in small volumes will depend on the spatial resolution of the SPECT. Small volumes of uptake may not be revealed correctly or be disguised by noise. Hence, the clinical scanner cannot be used to conclude that there is little uptake in small volumes, such as mouse organs, and it might give a false indication of a favourable biodistribution. Whilst the same principles apply for the preclinical SPECT system, the superior image quality allows for seeing uptake in smaller mouse organs.

## Data Availability

The datasets from this study are available upon request.

## Abbreviations

PSMA: Prostate specific membrane antigen
HE: High energy
ME: Medium energy
